# Understanding the phenotype of genetically associated electronegative ERG retinopathies: comparing the full-field ERG b:a ratio

**DOI:** 10.1007/s00417-025-06851-4

**Published:** 2025-05-29

**Authors:** Christopher A. Ovens, Elisa E. Cornish, Haipha Ali, Vannessa Leung, Dhimas H. Sakti, Nonna Saakova, Marium Raza, Benjamin M. Nash, Clare L. Fraser, Peter McCluskey, Robyn V. Jamieson, John R. Grigg

**Affiliations:** 1 Save Sight Institute, Faculty of Medicine and Health, The University of Sydney, Sydney, NSW Australia; 2 Eye Genetics Research Unit, Children’s Medical Research Institute, The Children’s Hospital at Westmead, Westmead, NSW Australia; 3 Department of Ophthalmology, Faculty of Medicine, Public Health, and Nursing, Universitas Gadjah Mada, Yogyakarta, Indonesia; 4 Sydney Genome Diagnostics, Sydney Children’s Hospital Network, Western Sydney Genetics Program, Westmead, NSW Australia

**Keywords:** Electronegative, Inherited retinal diseases, X-linked retinoschisis, Electroretinogram

## Abstract

**Purpose:**

The electronegative electroretinogram (ERG) is a specific clinical finding usually indicating inner retinal dysfunction occurring post-phototransduction. X-linked retinoschisis (XLRS) and complete and incomplete congenital stationary night blindness (cCSNB, iCSNB) are inherited retinal dystrophies classically associated with electronegative ERGs. Comparing the full-field ERG b:a ratio expands current ERG diagnostic criteria and aids in localising physiological sites and pathological mechanisms.

**Methods:**

A retrospective review of patients with a clinical diagnosis of iCSNB, cCSNB and XLRS was conducted. ERG and genetic results were analysed. Average b:a ratios between groups were compared, and prevalence of electropositivity was assessed using thresholds of b:a > 1.0 and b:a > 1.50.

**Results:**

53 patients were included, and genetic confirmation was available in 7/24 iCSNB, 3/14 cCSNB and 11/15 XLRS patients respectively. In genetically proven cases, mean b:a ratio in XLRS patients (b:a = 1.04) was significantly higher than cCSNB (b:a = 0.60, *p* < 0.001) and iCSNB (b:a = 0.60, *p* < 0.001). An electropositive ERG was significantly more likely to be associated with RS1 than iCSNB (*p* < 0.001) or cCSNB (*p* = 0.001) at b:a > 1.0 threshold, and more likely RS1 than iCSNB (*p* = 0.040) at b:a > 1.5 threshold.

**Conclusion:**

Our study highlights the distinct ERG findings between these typically electronegative inner retinal dystrophies. In a clinical setting, the traditional electronegative definition of b:a < 1.0 appears very insensitive to detect XLRS patients. Our data suggests clinical suspicion should remain even in patients with a b:a ratio > 1.50, and highlights the importance of genetic testing in these cases.

## Abstract (highlights)

**Key messages:**

*****What is known***:**

- An electronegative electroretinogram (ERG) (b:a wave ratio < 1.0) is an important clinical finding in several inherited retinal diseases, including complete and incomplete congenital stationary night blindness (cCSNB, iCSNB) and X-linked retinoschosis (XLRS).

*****What is new***:**

- A large number of our X-linked retinoschisis patients did not display an electronegative ERG and were associated with missense mutations, in contrast to iCSNB and cCSNB patients.
- When combining these results with recent molecular and functional studies, this offers new insights into the underlying pathophysiology of XLRS at the photoreceptor-bipolar synapse.
- Expanding our understanding of the underlying pathophysiology and phenotypic variability of classically electronegative retinal diseases helps to explain ERG findings, improves diagnostic accuracy and guides genetic testing and its interpretation.

## Introduction

An “electronegative” full-field electroretinogram (ffERG) has traditionally been defined as a b:a-wave ratio < 1.0, whereby b-wave amplitude is selectively reduced such that it is less than the preceding a-wave [1]. The electronegative ERG is a specific clinical finding that usually indicates inner retinal dysfunction occurring post-phototransduction, at the level of the photoreceptor synapse or bipolar cell. [1] This functional parameter is important in directing further investigations including genomic testing, and carries a small number of differentials.

Inherited retinal diseases (IRDs) classically associated with electronegative ffERGs include X-linked retinoschisis (XLRS), complete and incomplete congenital stationary night blindness (cCSNB, iCSNB) [2]. The full-field electroretinogram (ERG) is important for diagnosis of these conditions due to significant phenotypic variation. Each display post-transduction dysfunction manifesting as a selective reduction in bipolar cell depolarisation (b-wave) compared to photoreceptor hyperpolarisation (a-wave). [1]

However, we and others have identified that not all patients with the classically associated IRDs have an electronegative ERG by the standard definition [1, 3–5]. Studies have highlighted significant heterogeneity in the ffERG findings within and between these three conditions reflecting variations in genotype and subsequent downstream alterations to the transduction cascade [3–5]. In particular, up to 80% of XLRS patient have been reported to be electropositive using a definition of b:a-wave > 1.0 [4, 6–8]. A careful characterisation of this biomarker is required to ensure detailed phenotyping and appropriate sensitivity for clinical practice. This in turn helps inform clinical genetics assessment, which is now standard-of-care for IRDs. In this study we assess the full-field ERG b:a wave ratio phenotypes of genetically confirmed IRD’s to evaluate whether the traditional “electronegative” b:a-wave criteria should be expanded.

## Methods

A retrospective review of patient files at Save Sight Institute, Sydney, Australia from 2008–2022 was performed. Patients with a clinical and electrophysiological diagnosis of iCSNB, cCSNB or XLRS were included. This diagnosis was based on careful history, physical examination, electrophysiology findings, and various structural and functional ancillary tests as appropriate. The cohort was further divided by identified genomic variant. Baseline characteristics including age and gender, as well as any known genetic mutation and inheritance pattern were collected.

### Genomic assessment

Next Generation Sequencing (NGS) was performed using exome sequencing (ES) with Illumina TruSight One Clinical Exome (Illumina, USA) or Agilent SureSelect Exome (Agilent SureSelect V4, Macrogen Inc, Seoul, SouthKorea). Associated disease genes were targeted, and the results interpreted by the clinical genetics service. Where a genetic diagnosis was available in XLRS patients, each mutation was further grouped as either Group A (missense or in-frame deletions) or Group B (nonsense, splice-site, or frame-shifting insertions or deletions) as per Vincent et al. [9]. Those patients without genetic confirmation are either awaiting genetic testing results, or have declined testing. Patients who have had genetic testing completed and who did not return a result consistent with the diagnosis have been excluded.

### Electrophysiology testing

Electrophysiology was performed using full-field ERGs according to the International Society for Clinical Electrophysiology of Vision (ISCEV) standards [10]. We focused on dark-adapted (DA) responses to 3.0 cd.s.m^−2^ (DA 3.0) and 12.0 cd.s.m^−2^ (DA 12.0) flash intensities to assess b wave and a wave amplitude. The ratio of the b wave to a wave (b:a ratio) under both DA 3.0 and DA 12.0 conditions was calculated for each eye of each patient.

Genetically proven cases of each diagnosis were used in all statistical analysis. Initially, mean b:a ratios of genetically confirmed iCSNB, cCSNB and XLRS were compared both to the overall “clinical diagnosis” cohort, as well as between different diagnoses. Results were taken from both eyes given the potential asymmetry of results particularly in XLRS. Ratios were evaluated separately for DA3.0 and DA 12.0 stimuli and were then compared, both using global mean b:a ratios for the cohort as well as paired b:a values for each eye of each participant. We then combined DA3.0 and DA 12.0 results to create “pooled” data. Comparison of b:a ratios between Group A and Group B of gen-XLRS patients was also made. Different cut-offs of electronegativity were also analysed. We ran parallel testing of two different definitions of an “electropositive ERG” according to b:a ratio – initially with the classic definition of b:a > 1.00, and again with a higher threshold of b:a > 1.50. We considered both individual traces (“trace analysis”) as well as a per-patient analysis (“patient analysis”). Statistical analysis was performed using Mann–Whitney U test for non-parametric data to compare b:a ratios within and between groups. Chi-squared testing was used to assess incidence of an electropositive ERG between groups. Significance level was set at *p* < 0.05.

## Results

We identified 53 patients who following electrophysiology were diagnosed with either iCSNB, cCSNB or XLRS. Demographic data is shown in Table 1. In total there were 24 iCSNB patients, 14 cCSNB patients and 15 XLRS patients included. Of these, 7 iCSNB, 3 cCSNB and 11 XLRS patients had a genetically confirmed diagnosis (with 28 traces, 12 traces and 34 traces respectively). Genetically proven cases will be referred to with the “gen- “ prefix. Patients without genetic confirmation had either declined genetic testing, were lost to follow-up, or returned an inconclusive or non-diagnostic result from genetic testing.

**Table 1:** Baseline characteristics of included patients

Demographics	Genetically proven (whole cohort)
Cases (n)	
iCSNB	7 (24)
cCSNB	3 (14)
XLRS	11 (15)
Mean Age (years)	
iCSNB	19.3 (23.3)
cCSNB	14.3 (19.5)
XLRS	17.1 (24.3)
Proportion Male	
iCSNB	5/7 (18/24)
cCSNB	0/3 (9/14)
XLRS	11/11 (15/15)
Electrophysiological data	Mean (± st dev)
Mean b:a-wave ratio – DA3.0 (± st dev)	
iCSNB	0.58 (± 0.18)
cCSNB	0.53 (± 0.09)
XLRS	1.01 (± 0.40)
Mean b:a-wave ratio – DA12.0 (± st dev)	
iCSNB	0.62 (± 0.20)
cCSNB	0.66 (± 0.16)
XLRS	1.07 (± 0.48)
Mean b:a-wave ratio – pooled (± st dev)	
iCSNB	0.60 (± 0.19)
cCSNB	0.60 (± 0.14)
XLRS	1.04 (± 0.44)
Genetic diagnosis	Gene
iCSNB	*CACN1 F (n = 6)*
	*CABP4* (*n* = 1)
cCSNB	*GPR179 (n = 2)*
XLRS	*TRPM1* (*n* = 1)
	*RS1* (*n* = 11)*

Legend: "Gen-” prefix denotes genetically-proven cases. *iCSNB* incomplete congenital stationary night blindness, *cCSNB* complete congenital stationary night blindness, *XLRS* x-linked retinoschisis. *RS1 mutations further characterised below

**Fig. 1:**
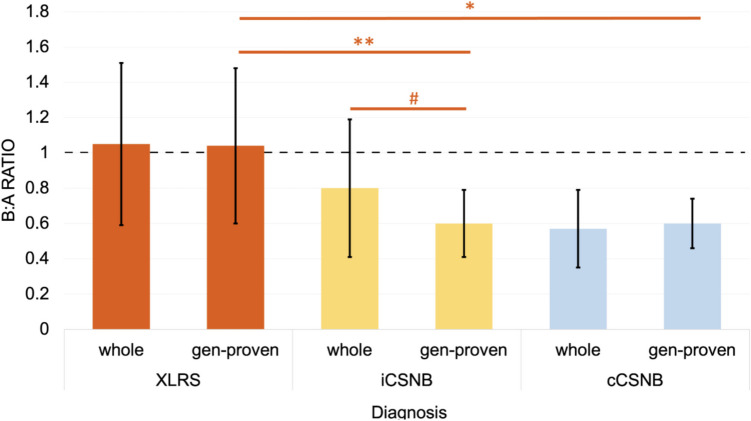
Mean pooled b:a ratios for each group. Significant differences between gen-XLRS patients compared to gen-cCSNB (*) and gen-iCSNB (**) patients are shown. The significantly lower b:a ratio seen in gen-iCSNB compared to the overall iCSNB cohort is also noted (#). Error bars denote 1 standard deviation. Horizontal broken line represents b:a ratio of 1. “Gen-” prefix denotes genetically-proven cases. iCSNB incomplete congenital stationary night blindness, cCSNB complete congenital stationary night blindness, XLRS x-linked retinoschisis

**Fig. 2:**
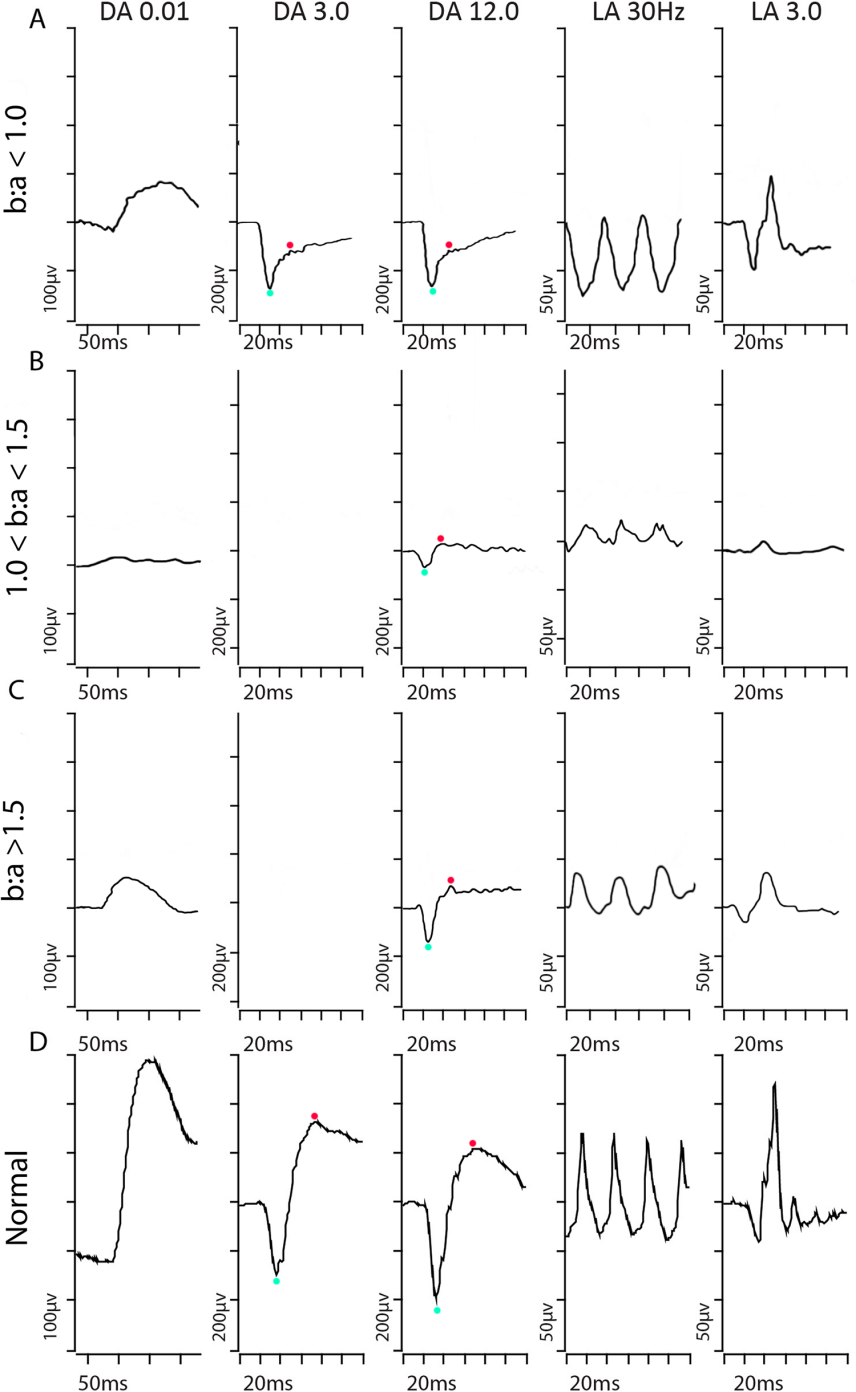
Sample traces from three *RS1* patients. Measurements for a-waves and b-waves were taken at light blue and red markers respectively. Row A shows an *RS1* patient with more classical electronegative dark-adapted responses to DA 3.0 and DA 12.0. Row B shows an *RS1* patient with b:a-wave ratio between 1.0 and 1.5 for DA 12.0 stimulus. Row C shows a third *RS1* patient with b:a ratio > 1.5 to DA 12.0 stimulus. These are compared to normal electrophysiological traces in Row D. In the ffERG the a-wave amplitudes are measured from baseline to a-wave trough, and b-wave amplitudes from a-wave trough to b-wave peak. The ffERG is termed electronegative if the b:a ratio is ≤ 1.0. The blue dot indicates the a-wave trough and red dot the b-wave peak

Mean b:a ratios for each group is shown in Fig. 1. In the iCSNB group mean b:a ratio was 0.80, while mean b:a ratio in gen-iCSNB cases was 0.60. The gen-iCSNB group had a significantly lower mean b:a ratio compared to the overall iCSNB group (*p* = 0.009). In the cCSNB group mean b:a ratio was 0.57, while in gen-cCSNB mean b:a ratio was 0.60. In the XLRS group mean b:a ratio was 1.05, while in gen-XLRS mean b:a ratio was 1.04. Sample traces can be visualised in Fig. 2. When DA 3.0 traces and DA 12.0 traces were compared, there was no significant difference between DA 3.0 and DA 12.0 results in any group in either the global or paired analysis. When DA 3.0 and DA 12.0 traces were pooled together (see Fig. 1), we found gen-XLRS b:a ratios to be significantly greater than both gen-iCSNB (*p* < 0.001) and gen-cCSNB (*p* < 0.001). There was no significant difference between gen-iCSNB and gen-cCSNB under any conditions. In our XLRS cohort, there were 3 patients who only had DA 12.0 traces (without DA 3.0), and two patients who had DA 3.0 traces (without DA 12.0).

**Fig. 3:**
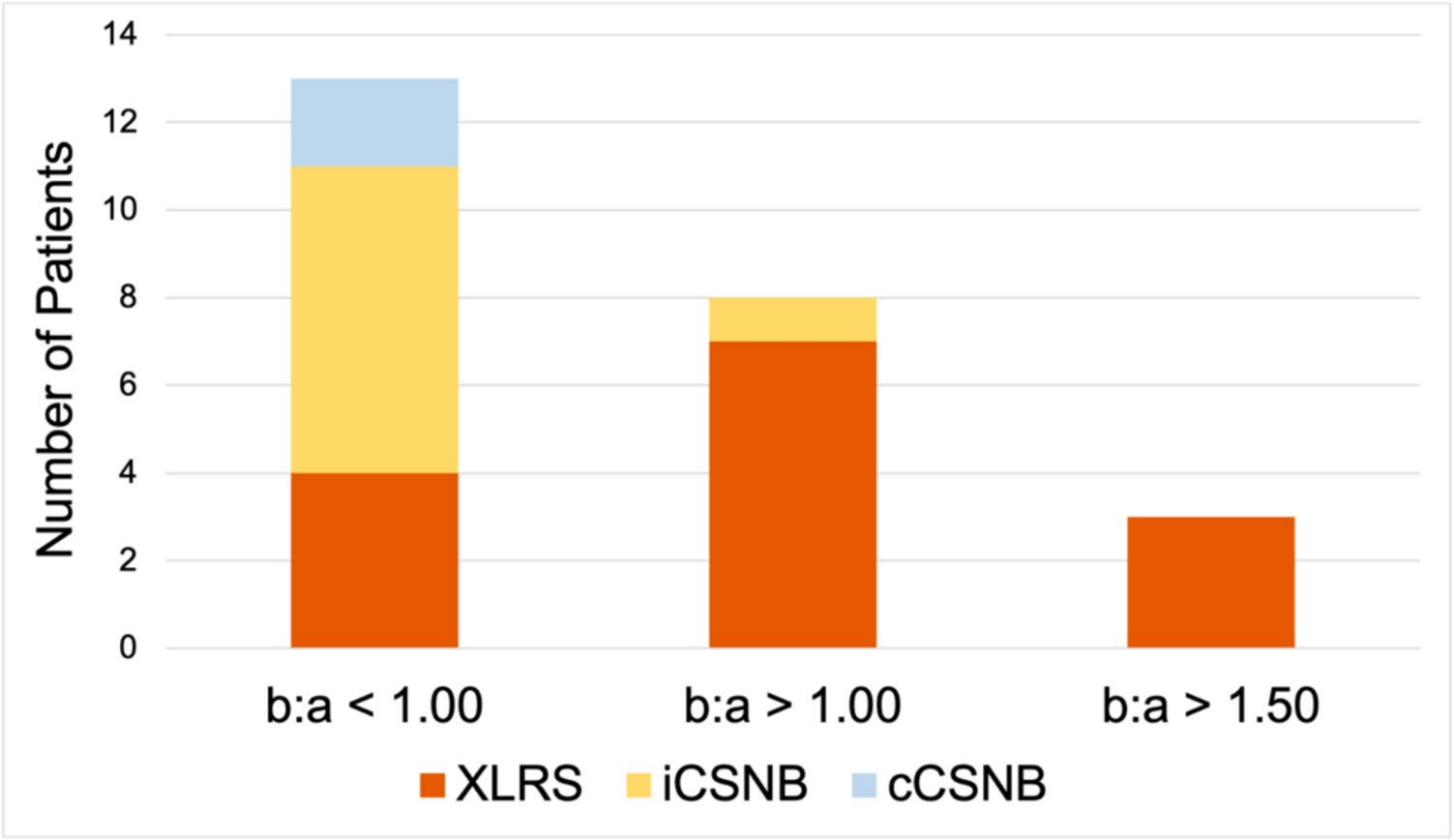
Spread of genetically-proven patients at differing electronegativity thresholds. With a threshold of b:a > 1.00, we saw both gen-XLRS and gen-iCSNB patients return electropositive results. Using a threshold of b:a > 1.50, only XLRS patients were found to have an electropositive result

The incidence of electropositive ERGs was calculated in genetically proven patients at b:a > 1.00 and b:a > 1.50 thresholds, as depicted in Fig. 3. No patients in the gen-cCSNB group had an electropositive trace, while 1 gen-iCSNB patient had an electropositive trace under DA12.0 conditions. Rates of electropositivity in the gen-XLRS group was higher under all conditions. Initial analysis was performed defining electropositivity as b:a > 1.00. When considering “trace analysis” of gen-XLRS, 8/16 (50%) of DA 3.0 and 10/18 (56%) of DA 12.0 traces were electropositive, which was significantly greater than iCSNB and cCSNB under the same conditions. In the “patient analysis” of gen-XLRS, incidence of electropositive ERG was, 3/8 under DA 3.0 conditions and 6/11 under DA 12.0 conditions. Again, this was significantly greater proportion than iCSNB (*p* = 0.040).

This analysis was repeated using a threshold of b:a > 1.50 to define “electropositivity”. In gen-XLRS traces, 2/16 (13%) of DA 3.0 traces and 3/18 (17%) of DA 12.0 traces were electropositive, which was significantly greater than iCSNB when pooled data was used (*p* = 0.024). In the patient analysis of gen-XLRS, incidence of electropositive ERG was 1/8 under DA 3.0 conditions, and 2/11 under DA 12.0 conditions. Gen-XLRS patients with *all* available traces being electropositive made up 5/11 patients at b:a > 1.00 threshold, and 2/11 patients at b:a > 1.50.

Genetic mutations were characterised for each condition. In gen-iCSNB, mutations were found in *CACN1 F* (*n* = 6) and *CABP4* (*n* = 1) genes, while in gen-cCSNB mutations were found in the *GPR179* (*n* = 2) and *TRPM1* (*n* = 1) genes. In gen-XLRS, patients were further characterised into two groups of genetic severity of *RS1* mutation (see Table 2). Group A (missense or in-frame deletions) comprised 5/11 patients and Group B (nonsense, splice-site, or frame-shifting insertions or deletions) comprised 6/11 patients. Mean age at ERG in Group A was 19.8 yrs and Group B was 14.8 yrs. There was no significant difference in age between groups (U = 124.0, *p* = 0.50). Mean b:a ratio in Group A was 1.18 and in Group B was 0.92. Group B was found to have significantly lower mean b:a ratio when compared to Group A (U = 75.5, *p* = 0.019). There was no significant difference in mean a-wave amplitude between the two groups.

**Table 2:** Genetic mutations in XLRS patients

Patient	Age	Mutation	Nature of mutation	Group
1	16	c.638G > T, p.Arg213Leu	Missense	A
2	10	c.638G > T, p.Arg213Leu	Missense	A
3	22	c.608 C > T, p.Pro203Leu	Missense	A
4	8	Exon 1–4 deletion in RS1	Frame-shift deletion	B
5	6	Exon 1–4 deletion in RS1	Frame-shift deletion	B
6	25	Exon 3 deletion in RS1	Frame-shift deletion	B
7	20	Exon 3 deletion in RS1	Frame-shift deletion	B
8	22	Exon 3 deletion in RS1	Frame-shift deletion	B
9	47	c.574 C > T, p.Pro192Ser	Missense	A
10	8	c.52 + 1G > T	Splice-site	B
11	4	c.590G > A, p.Arg197His	Missense	A

## Discussion

Dark adapted ISCEV standard ffERG traces [10] allow an evaluation of photoreceptor hyperpolarisation (negative a-wave) and post transduction inner retinal bipolar cell depolarisation (positive b-wave). Traditional measures have defined an electronegative waveform as one where the b-wave is smaller than the a-wave i.e. b:a < 1.0. This has been identified in 3.1–4.8% of all ERGs in large cohort studies [1, 11] and has a relatively finite group of differential diagnoses. iCSNB, cCSNB and XLRS are common causes, while other IRDs such as rod-cone dystrophy, cone-rod dystrophy and enhanced S-cone syndrome may also display an electronegative ERG. [1] CSNB is a heterogenous group of inherited retinal disorders with abnormality of signal processing at the level of the photoreceptor-bipolar synapse. The complete form (cCSNB) involves post-synaptic ON-bipolar pathway dysfunction with no detectable rod b-wave, and is commonly associated with mutations in *NYX*, *TRPM1*, *GRM6* or *GPR179* genes [2, 4, 12] The incomplete form (iCSNB) affects both ON- and OFF- cone bipolar pathways with some detectable rod function, with mutations in *CACNA1 F*, *CABP4* and *RIMS2* genes all described [2, 12, 13]. XLRS is caused by mutation in the *RS1* gene encoding retinoschisin, an extracellular cell-adhesion molecule secreted primarily by photoreceptors and bipolar cells [14]. It is located at pre-synaptic photoreceptor and post-synaptic bipolar cell sites which governs molecular synaptic transmission [15]. Electronegativity varies according to the type of mutation (eg missense vs other) as well as disease severity [6, 9], however the exact mechanism of inner retinal dysfunction in XLRS remains unclear.

**Fig. 4:**
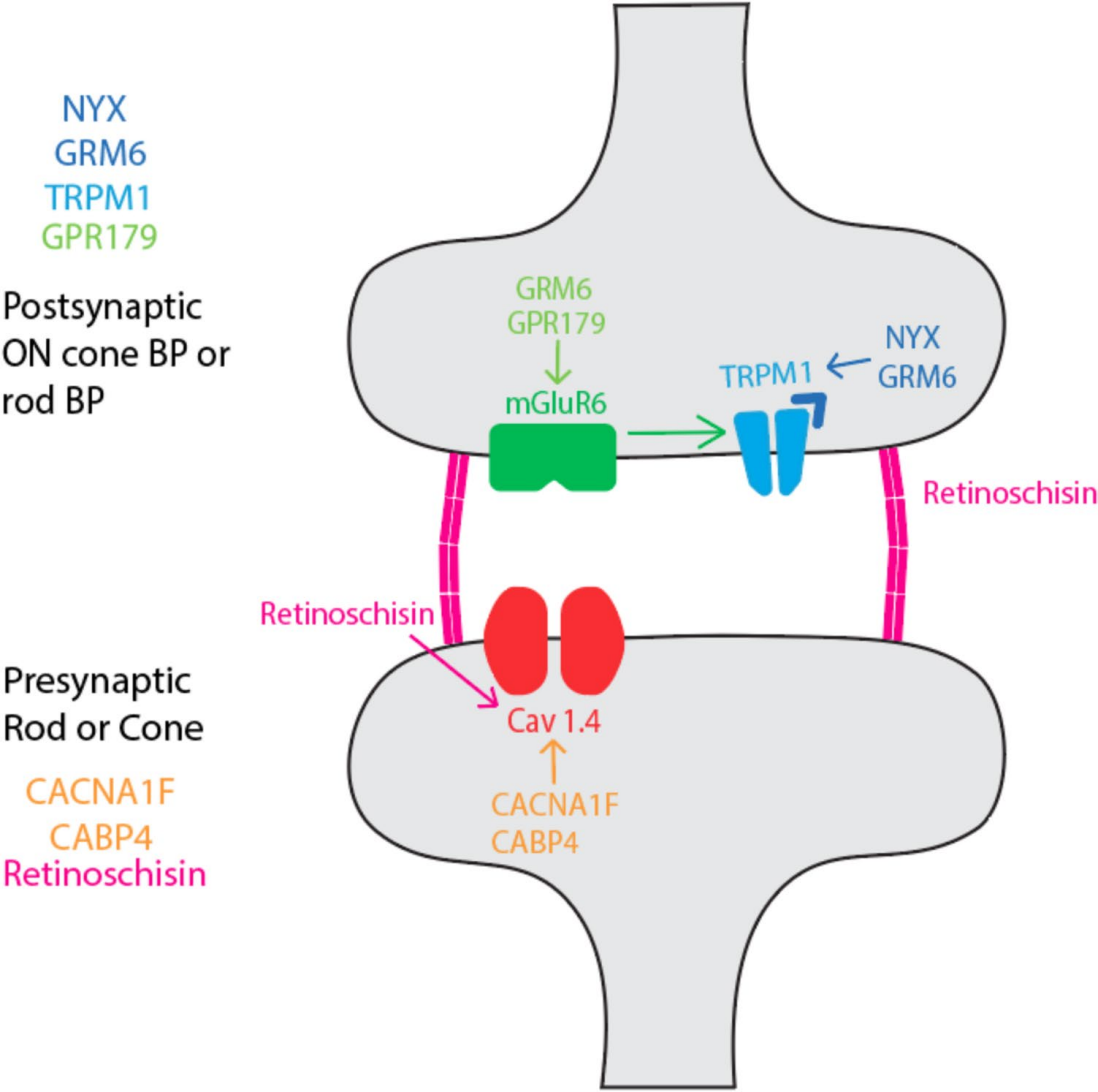
Schematic of gene protein locations and interactions at the photoreceptor-bipolar cell synapse. Arrows denote a vector of effect, for example “X → Y” means X affects Y, and dysfunction of X will result in dysfunction of Y. Pre-synaptic cation channel Cav1.4 is regulated by *CACNA1 F* and *CABP4*. Mutations in *CACNA1 F* and *CABP4* genes lead to iCSNB via interaction with Cav1.4 channel. It is now understood that in addition to providing cell–cell adhesion and support, retinoschisin also regulates the presynaptic cation channel Cav1.4. Post-synaptic mGluR6 is regulated by *GRM6* and *GPR179* genes, while *NYX* and *GRM6* are critical in localization of TRPM1 at the synapse. Dysfunction of *NYX*, *GRM6*, *TRPM1* and *GPR179* lead to cCSNB. This illustrates how *RS1* mutations in retinoschisin may result in a similar phenotype to *CACNA1F*-mutant iCSNB patients by implicating a common pathological pathway

In our patients, electronegativity and b:a ratios differed between groups. All gen-cCSNB patients had electronegative traces, with a mean b:a ratio of 0.60. The post-synaptic ON-bipolar disruption associated with *GPR179* (*n* = 2) and *TRPM1* (*n* = 1) mutations in our patients are known to cause significantly attenuated dark-adapted bipolar responses [2]. *TRPM1* dysfunction limits cation influx with light stimulus, thereby reducing bipolar depolarisation and signal transduction [12]. It is thought that GPR179 colocalizes and interacts with GTPase accelerating proteins involved in mGluR6-dependent signalling (see Fig. 4) [12]. The vast majority reported in the literature are associated with *NYX* mutation and have an electronegative ffERG which is consistent with our findings [4].

Our gen-iCSNB cohort displayed a similar phenotype with several exceptions. The significant difference in mean b:a ratio between gen-iCSNB and the overall iCSNB cohort (0.60 vs 0.80, *p* = 0.009) highlights that a phenotypic diagnosis in inherited retinal diseases is often insufficient. Incompletely understood genetic heterogeneity and genotype–phenotype correlations may explain these findings, and may offer alternative diagnoses for some of these iCSNB patients. The corollary is that b:a ratios in those with a clinical phenotype of XLRS or cCSNB shows good agreement with b:a ratios of genetically confirmed cases. In our gen-iCSNB group, mean b:a ratio was also electronegative (b:a = 0.60). We identified mutations in *CACNA1F* and *CABP4*, both previously reported to reduce Cav1.4 levels and functioning at presynaptic photoreceptor terminals (see Fig. 4) [16]. Notably, one iCSNB patient with *CACNA1F* displayed an electropositive ERG. This patient had b:a ratios in the right and left eyes of 0.9 and 0.98 respectively under DA 3.0 stimulus, and 1.1 and 0.9 respectively under DA 12.0 stimulus. Usually the brighter flash will result in a smaller b:a ratio [10], although we found no significant difference between DA3.0 and DA 12.0 responses in our cohort. This patient also has a younger brother who is symptomatic but has not yet had genetic testing, in whom only 1/4 of the equivalent traces were electronegative.

The most notable variation in electronegative traces was seen in our gen-XLRS patients. Gen-XLRS patients had a significantly higher mean b:a ratio than both iCSNB and cCSNB under all conditions. In fact, mean b:a ratio was electropositive (b:a = 1.04) in our XLRS cohort, with 18/34 (53%) traces being electropositive. This highlights the variability of b:a ratios in this patient group, in keeping with previous studies which found between 0–80% of XLRS patients to be electropositive [4, 6–8]. This heterogeneity has been linked to the type of mutation (eg missense vs other) as well as disease severity [6, 9], however the exact mechanism of inner retinal dysfunction in XLRS remains unclear. At a molecular level, Sergeev et al. found a relationship between the predicted structural severity of retinoschisin mutation with the b wave and b:a ratio in XLRS patients [17]. Mutations involving cysteine residues or the hydrophobic core of RS1 were particularly disruptive to protein structure. Functionally, Shi et al. [18] have shown an interaction between retinoschisin and the *CACNA1F*-encoded voltage-gated calcium channel Cav1.4, responsible for glutamate release at the photoreceptor-bipolar cell synapse. Rs1 was found to enhance Cav1.4 activation and current density, while knockout Rs1-/- mice models showed reduced Cav1.4 in the retinal outer plexiform layer. This suggests both structural and functional interaction between retinoschisin and Cav1.4, and may explain the electronegative ffERG found in XLRS. *CACNA1F *mutations in iCSNB also affect the Cav1.4 channel [16], and the b-wave amplitudes have elsewhere been found to be similar between *CACNA1F*-related iCSNB and XLRS groups [4]. Both were affected less than *NYX*-related cCSNB in this study, while a-wave amplitudes were reduced in *RS1* but not *CACNA1F* patients consistent with the more widespread role of retinoschisin as noted in other studies [4, 17]. Thus, we propose the highly variable and more electropositive ffERG findings in XLRS compared with iCSNB is due to Cav1.4-mediated b-wave reduction [18] combined with more widespread retinoschisin-mediated a-wave reduction [4]. As to the magnitude of b wave and a wave disruption and the resulting b:a ratio, this may be related to the mutation-specific severity of structural retinoschisin disruption [17].

The variability of b:a ratios in XLRS patients requires further comment. When divided by mutation type as per Vincent et al. [9], we also found a significantly lower b:a ratio in patients with “more severe” mutations. Severe mutations (Group B: nonsense, splice-site, or frame-shift, mean b:a = 0.92) caused significantly lower b:a ratios compared to less severe mutations (Group A: missense or in-frame deletions, mean b:a = 1.15, see Table 2). This observation has been reported by Bowles et al. [6] and Vincent et al. [9] and may partly explain the variation in electronegativity rates reported in the literature. We also found no difference in a wave amplitudes between these groups, suggesting that the difference in b:a ratio is attributable to the post-transduction b-wave component. There is conflicting evidence regarding ERG deterioration with age, however our data found no significant association, in agreement with Vincent et al. [9]. In a clinical setting, this makes the traditional electronegative definition of b:a < 1.0 very insensitive to detect XLRS patients. Our data suggests clinical suspicion should remain even in patients with a b:a ratio > 1.50. In our RS1 patients, 18% had b:a ratios > 1.50 across all stimuli which highlights the importance of clinical assessment and genetic testing in unexplained or borderline cases. Electrophysiological examination in these conditions should not occur in isolation but should form part of a multi-modal assessment to assist diagnosis and phenotypic description [19]. This is evident in cases of similar phenotypes such as with CRB1 retinopathy [20] where cystic and schitic macular changes are also present. In these cases the ffERG may help to distinguish overlapping phenotypes and aid genetic investigations. In the era of widely available genomic testing, this should be sought early in the patient’s diagnostic journey. However, genetic testing may be delayed or inconclusive such as in the case of variants of uncertain significance. Thus, a nuanced understanding of ERG findings in these IRDs enables accurate phenotyping to complement and inform genetic testing.

Our results are limited by relatively small patient numbers (particularly in the gen-cCSNB group), and limitations inherent to all retrospective studies such as incomplete data sets and updated testing procedures and equipment are also present here. Some patients did not have complete ERG traces (eg DA 12.0 only). Others did not have a confirmed genetic diagnosis, either due to patients declining genetic testing, being lost to follow-up, or for whom the results were not diagnostic or inconclusive.

## Conclusions

These findings highlight the expanded variability in b:a ratios. Patients with genetically proven disease in some cases exhibited a positive b:a ratio. This work suggests that a b:a ratio up to at least 1.5 does not exclude an IRD that classically has been grouped as an electronegative condition. XLRS patients were frequently found to be electropositive. All CSNB cases illustrated a b:a ratio of < 1.50, and all cCSNB cases had a ratio < 1.0. The variation in ERG b:a ratio between the groups further assists in guiding genetic testing and its interpretation.
